# A rare mediastinal-type superior vena cava cavernous hemangioma successfully treated with surgical resection under cardiopulmonary bypass condition: a case report

**DOI:** 10.3389/fcvm.2026.1722316

**Published:** 2026-01-27

**Authors:** Qun-Xian Zhang, Ye Yang, Tao Liu, Jun Zhang, Qiang Guo, Hua Liu, Min Zeng, Dan Li

**Affiliations:** 1 Department of Cardiothoracic Surgery, Taihe Hospital, Hubei University of Medicine, Shiyan, Hubei Province, China; 2 Department of Psychiatry, Jingwei Hospital of Shiyan, Shiyan, Hubei Province, China; 3 Department of Oncology, Taihe Hospital, Hubei University of Medicine, Shiyan, Hubei Province, China

**Keywords:** case report, cavernous hemangioma of the superior vena cava, chest CT scan, mediastinal tumor, thymoma

## Abstract

Of the anterior mediastinal tumors, cavernous hemangiomas originating from the superior vena cava (SVC) in this region are rare. We present a case in which the chest CT scan during a health check-up revealed an irregular, patchy soft-tissue density shadow in the thymic region, measuring approximately 5.5 × 3.5 cm in diameter. Eight months later, an enhanced chest CT scan at our hospital demonstrated an irregular, mass-like soft-tissue density shadow in the anterior mediastinum, with poorly defined borders adjacent to vascular structures and an increased diameter of approximately 6.0 × 3.8 cm. During surgery, the lesion exhibited a hemangioma-like appearance, with its base located at the confluence of the left and right innominate veins into the SVC. Mediastinal lesion resection and SVC plasty were therefore performed under cardiopulmonary bypass. Postoperative pathological examination confirmed the diagnosis of SVC cavernous hemangioma. The patient recovered uneventfully and was discharged on postoperative day 6. A follow-up chest CT scan more than three months after surgery showed imaging changes consistent with resection of the cavernous hemangioma. In addition, atelectasis in the right middle and lower lobes had significantly improved compared to earlier imaging. This case offers valuable clinical experience and insights to support treatment decision-making in similar scenarios.

## Background

Common anterior mediastinal tumors include thymoma, teratoma, lymphoma, and thymic carcinoma (1, 2). While minimally invasive resection is often the preferred surgical approach for such tumors, a subset of patients may still require open thoracotomy. Cavernous hemangioma of the superior vena cava is a rare entity that poses significant surgical challenges (3). We report the case of a patient whose preoperative contrast-enhanced chest CT revealed an irregular soft-tissue mass in the anterior mediastinum, measuring approximately 6.0 × 3.8 cm, with ill-defined borders adjacent to vascular structures. Intraoperatively, the lesion was found to originate from the confluence of the left and right brachiocephalic veins into the superior vena cava. The resection was performed under cardiopulmonary bypass (CPB) to ensure patient safety. The patient had an uneventful recovery and was discharged on the sixth postoperative day. This case offers valuable insights to support clinical decision-making in the management of such rare vascular tumors.

## Case description

**Figure 1:**
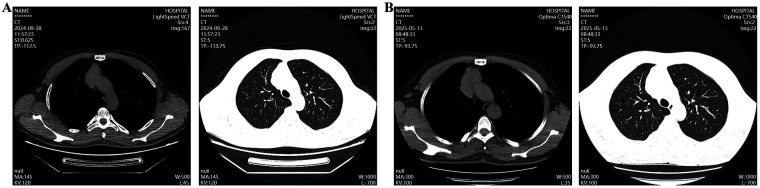
Chest CT scan revealed a mediastinal space-occupying lesion. **(A)** The first chest CT scan; **(B)** A chest CT scan after eight months.

The patient is a 40-year-old male. Eight months prior to admission, chest CT scan performed during a health checkup at our hospital revealed an irregular, patchy soft-tissue density shadow in the thymic region of the anterior mediastinum. The lesion measured approximately 5.5 × 3.5 cm in diameter, with ill-defined borders adjacent to blood vessels (Figure 1A). The patient reported no symptoms such as eyelid weakness, facial or upper extremity swelling, or jugular venous distension. No intervention was undertaken at that time. Eight months later, a follow-up chest CT demonstrated an irregular, patchy soft-tissue density shadow in the anterior mediastinum, again with poorly defined margins abutting adjacent vessels. The lesion had increased in size, measuring approximately 6.0 × 3.8 cm in diameter (Figure 1B).

**Figure 2:**
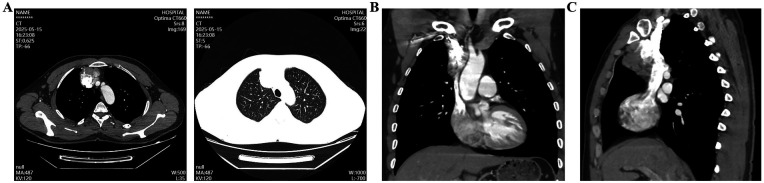
Enhanced chest CT revealed a mediastinal space-occupying lesion. **(A)** Mediastinal window and lung window; **(B,C)** Coronal and sagittal positions.

**Figure 3:**
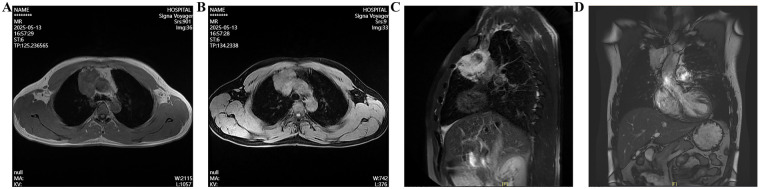
Chest magnetic resonance imaging shows a space-occupying lesion in the anterior mediastinum. **(A–D)** Phenomena of magnetic resonance at different periods and levels.

The patient denied any history of hypertension, diabetes, coronary heart disease, or hyperlipidemia. Physical examination on admission: The patient's vital signs were recorded as follows: temperature 36.3 °C, heart rate 72 bpm, respiratory rate 18 bpm, and blood pressure 126/86 mmHg. The chest was symmetrical with no deformities, and breath sounds were clear bilaterally without wheezes or crackles. The precordium showed no bulging or thrills; heart sounds were regular and strong, with no murmurs. There was no edema in the lower extremities. After admission, enhanced chest CT confirmed an irregular mass-like soft-tissue density shadow in the anterior mediastinum, with indistinct borders adjacent to vascular structures and increased dimensions compared to prior imaging, measuring approximately 6.0 × 3.8 cm (Figures 2A–C). Chest Magnetic resonance imaging (MRI) demonstrated a patchy abnormal signal in the thymic region, measuring about 3.9 × 6.3 × 5.6 cm, with relatively well-defined margins. The lesion showed high signal intensity on T2-weighted images and low signal on T1-weighted images, with partial borders indistinct from adjacent vessels. Major cardiac vessels exhibited normal flow voids (Figure 3). Echocardiography revealed left atrial enlargement, mild mitral regurgitation, and an ejection fraction of 65%. On May 26, 2025, the patient underwent mediastinal lesion resection and superior vena cava (SVC) plasty under CPB.

## Surgical procedure

**Figure 4:**
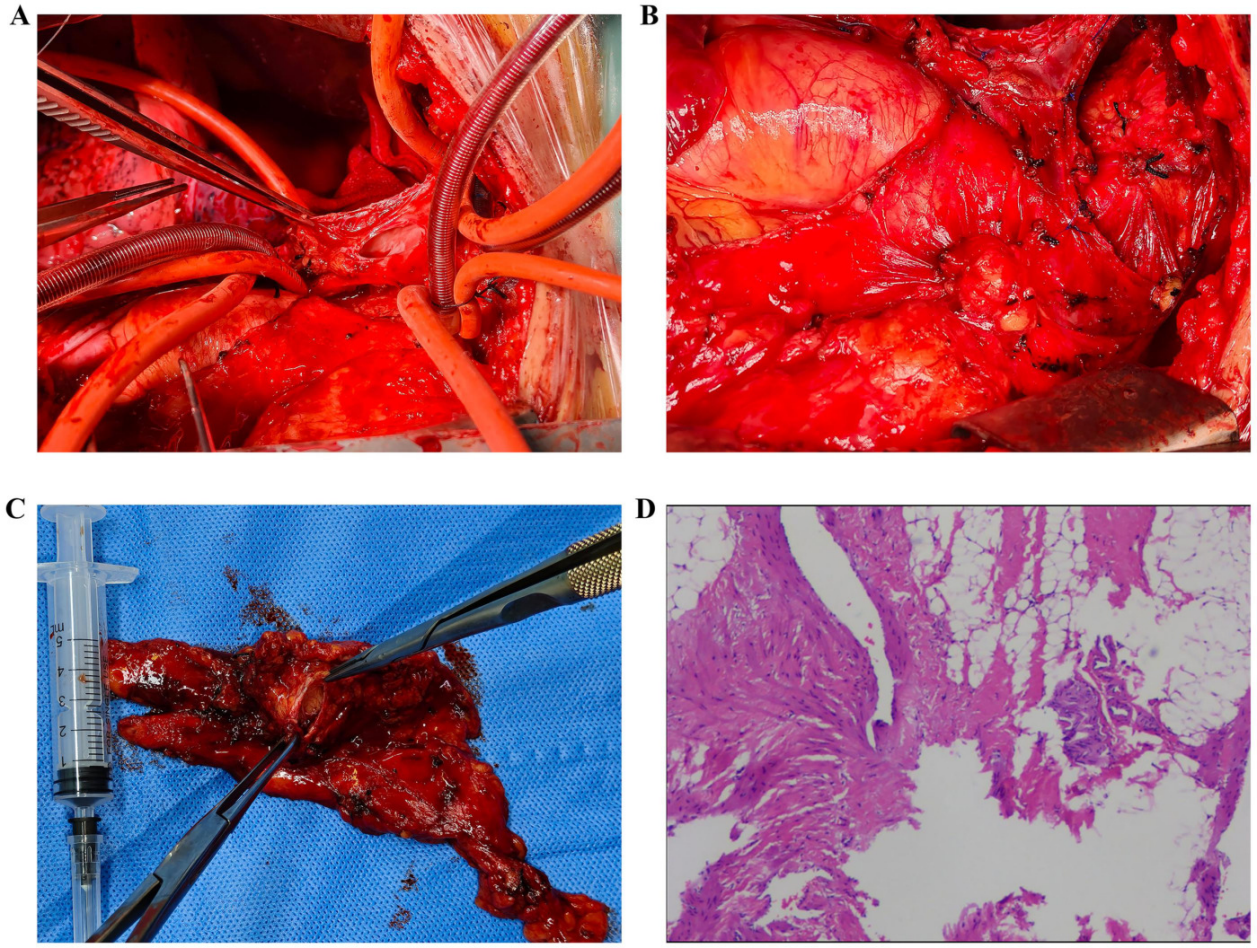
Intraoperative conditions and postoperative pathological results. **(A,B)** The location of the lesion and after the lesion surgery; **(C)** Lesion removed by surgery; **(D)** Postoperative pathological results.

Under general anesthesia, a median sternotomy was performed. The anterior mediastinal lesion appeared hemangioma-like, with a surface covered by tortuous, fragile vessels that bled easily on contact. The lesion completely encased the phrenic nerve. Further dissection confirmed that the base of the lesion was situated at the confluence of the left and right brachiocephalic veins into the SVC, with shared blood flow between the lesion and the venous system (Figures 4A–C). Following systemic heparinization, the superior vena cava proximal to the hemangioma was cannulated and connected to the CPB circuit. Once on full CPB, the venous return from the left and right brachiocephalic veins was diverted into the superior vena cava distal to the lesion. This allowed for the occlusion of the left and right brachiocephalic veins and the azygos vein to prevent retrograde flow, thereby facilitating the subsequent therapeutic procedure. Upon incising the lesion, malformed vascular channels were observed inside, with one opening communicating to the SVC origin measuring approximately 2 × 1 cm. Following the complete resection of the abnormal vasculature, the surgical defect was reconstructed with an autologous pericardial patch. After de-airing and releasing the clamps, venous pressures were measured and found to be satisfactory. Venous cannulas were removed, and protamine was administered to reverse heparinization. The CPB time was approximately 50 min. Hemostasis was secured with gauze packing and biological fibrin glue. A pericardial drain and a right pleural drain were placed, and the incision was closed.

**Figure 5:**
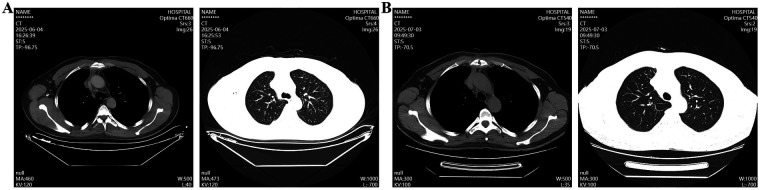
The chest CT scan after the operation indicated that the postoperative condition was good. **(A)** The results of a plain chest CT scan in June 4, 2025; **(B)** The results of a plain chest CT scan in July 3, 2025.

The resected tissue was visible to the naked eye as grayish-yellow irregular tissue, with a size of 7 × 6.5 × 2 cm. Some areas of the section show honeycomb-like changes, with a size of 2.5 × 2.5 × 1.7 cm. The remaining section is grayish-yellow, solid, soft and lobulated. Postoperative pathological examination confirmed the diagnosis of SVC cavernous hemangioma (Figure 4D). A non-contrast chest CT showed postoperative changes consistent with resection of the anterior mediastinal lesion (Figure 5A). The patient recovered well and was discharged on postoperative day 6. A follow-up CT more than three months after surgery revealed expected post-resection changes; atelectasis in the right middle and lower lobes had significantly improved compared to earlier studies (Figure 5B).

## Discussion

Mediastinal space-occupying lesions may arise from various tissues and organs within the mediastinum or represent metastases from distant sites. The clinical presentation of these lesions is highly variable and depends on factors such as size, location, pathological characteristics, and relationship with adjacent structures. Most patients are asymptomatic in the early stages, with lesions often incidentally detected on non-contrast chest CT. MRI can provide additional diagnostic information in selected cases. In the present case, chest CT performed during a health checkup revealed an irregular, patchy soft-tissue density shadow in the thymic region of the anterior mediastinum, measuring approximately 5.5 × 3.5 cm, with ill-defined borders adjacent to blood vessels. Although thymoma was considered highly likely, the patient exhibited no symptoms such as eyelid weakness and had no history of hypertension, diabetes, coronary heart disease, or hyperlipidemia. Enhanced CT and MRI of the chest demonstrated a well-circumscribed patchy abnormal signal in the thymic area. Based on the imaging features and clinical history, a benign tumor was suspected, consistent with findings reported in the literature.

With the exception of lymphoma, most mediastinal masses are managed surgically, often via thoracoscopic resection (4–6). For example, Yun et al. reported a case of posterior mediastinal cavernous hemangioma that had invaded and damaged the fifth rib. Fortunately, a total hemangioma resection was performed (4). Consistent with the findings of Yun et al., we concur that surgical intervention is the primary therapeutic option. Preoperative MRI revealed, however, that the lesion had ill-defined margins with adjacent vasculature, raising the possibility of a vascular tumor such as a hemangioma. However, imaging alone could not confirm the presence of vascular communication. The SVC has a thin wall and is under relatively high pressure, surrounded by critical structures including the pericardium, pulmonary artery, and phrenic nerve. Surgical manipulation in this area carries a significant risk of life-threatening hemorrhage. Therefore, ensuring patient safety during resection was a major consideration. Given the location, extent, and suspected vascular nature of the lesion, a median sternotomy with resection under CPB was deemed the safest and most effective approach. Intraoperatively, the lesion was identified as a venous cavernous hemangioma, characterized by a surface covered with fragile, malformed vessels that bled readily upon contact. The phrenic nerve was fully encased by the mass, and the base of the lesion was located at the confluence of the left and right brachiocephalic veins into the SVC, with shared blood flow between the lesion and the venous system. The surgical team adapted the strategy accordingly and successfully completed the resection under CPB.

SVC cavernous hemangioma is an exceptionally rare benign vascular tumor arising from the SVC wall. Its diagnosis is challenging due to its rarity and nonspecific imaging appearance. Enhanced CT and MRI scans are helpful for diagnosing hemangioma, but they still have certain limitations (7). Although the lesion in this case was asymptomatic, it caused compression on the brachiocephalic veins and their confluence, as seen on enhanced CT and MRI. Nevertheless, a definitive diagnosis could only be established by histopathological examination, which postoperatively confirmed SVC cavernous hemangioma, consistent with the intraoperative findings.

A limitation in this case was the inability to attempt a minimally invasive or smaller incision approach, which represents a focus for future technical refinement. In summary, resection of SVC cavernous hemangioma carries a high risk of bleeding and should be performed by an experienced cardiac or thoracic surgical team. The selection of an appropriate surgical strategy is central to the management of this condition. the management of such patients requires thorough preoperative evaluation and a cautious, individualized surgical plan. The objective of this case report is to share insights and experience in clinical diagnosis and management, aiming to assist frontline clinicians in managing similar cases.

## Data Availability

The original contributions presented in the study are included in the article/Supplementary Material, further inquiries can be directed to the corresponding authors.
